# Pectic hydrocolloids from steam‐exploded lime pectin peel: Effect of temperature and time on macromolecular and functional properties

**DOI:** 10.1002/fsn3.2158

**Published:** 2021-02-12

**Authors:** Randall G. Cameron, Elena Branca, Christina Dorado, Yang Kim

**Affiliations:** ^1^ United States Department of Agriculture Agricultural Research Service U.S. Horticultural Research Laboratory, Citrus and Other Subtropical Products Research Unit Fort Pierce FL USA; ^2^ Center for Food and Bioconvergence Seoul National University Seoul South Korea

**Keywords:** citrus, hydrocolloid, intrinsic viscosity, pectin, recovery, steam explosion

## Abstract

Previously, we showed the weight average molecular weight (*M*_w_) and intrinsic viscosity ([*ƞ*]) of pectic hydrocolloids recovered from steam‐exploded citrus peel were low, suggesting fragmentation due to process temperature and/or time‐at‐temperature. We have tested this hypothesis on a commercial lime pectin peel, washed to remove soluble sugars and dried for stabilization, using a static steam explosion system. We examined temperatures of 120–150°C at 1–3 min hold times. Galacturonic acid recovery and *M*_w_ ranged from 22% to 82% and 142–214 kDa, respectively. Recovery of most major pectic sugars increased concomitantly with galacturonic acid as temperature and time‐at‐temperature increased. [*ƞ*] ranged from 1.75 to 6.83 dl/g. The degree of methylesterification ranged from 66.5% to 72.1%. Tan (δ) (Loss modulus/Storage modulus; G″/G′) values of sugar–acid gels for 120–140°C treatments were <1.0. Ideal optimization analysis, where time, [*ƞ*], and percent recovery were maximized, identified processing conditions that favor either increased [*ƞ*] or percent recovery. The results presented here support our hypothesis that temperature and time‐at‐temperature affect *M*_w_ and [*η*] of the recovered pectic hydrocolloids. These results also demonstrate that manipulating either temperature or time‐at‐temperature enables the production of structurally varied populations of pectic hydrocolloids. Based on optimization analysis, commercially viable values of [*ƞ*] can be obtained while recovering approximately 50% of the pectic hydrocolloids.

## Graphical abstract

We demonstrate the effect of process temperature and time on the macromolecular and functional properties of pectic hydrocolloids obtained by steam explosion. Yield, molecular weight, intrinsic viscosity, and functional properties were affected by process conditions.

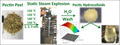

## 1 INTRODUCTION

Growth in the demand for pectin is reported to have been nearly 6% from 2010 to 2017. It is forecasted to maintain annual growth rates of almost 5% over the next several years (IMARC, 2018). This growth is expected to be highest in the dairy and jam/fruit application sectors (Technavio, 2018). Nearly 63.5 × 10^6^ kg of pectin were estimated to have been produced in 2017 (IMARC, 2018), of which approximately 85% is estimated to have been from citrus (Staunstrup, 2018). Despite this increasing demand for pectin, the potential supply far exceeds these enthusiastic predictions as approximately 10 × 10^6^ Mg of oranges for processing were forecast from Brazil and 3 × 10^6^ Mg from the United States for 2017–2018 (Anonymous, 2018) with a pectin content of approximately 2% of fruit fresh weight.

Pectin has been described as having a canonical structure (Pérez et al., 2000), suggesting it is organized along well‐established patterns. Pectin also is commonly considered to be a hetero‐block copolymer (Voragen et al., 2009; Williams et al., 2009). Pectin's two most common copolymers are the homogalacturonan (HG) region (α‐1,4 linked D‐GalA), and the rhamnogalacturonan I (RG I) region (McNeil et al., 1984). The RG I backbone is a repeating dimer of [→2)‐α‐L‐Rha*p*‐(1 → 4)‐α‐D‐GalA*p*‐(1→]_n_ (Voragen et al., 2009). Both neutral arabinan and (arabino‐) galactans may be attached to *O*‐4 of the rhamnose residues (Albersheim et al., 1996; Wefers et al., 2014). Supporting evidence has been provided that RG I regions are interspersed between HG regions (Coenen et al., 2007) and that the degree of polymerization for HG regions in citrus pectin is between 81 and 117 GalAs (Yapo et al., 2007). Non‐HG‐associated RG I has been reported from highly soluble fruit parenchyma cells (Cornuault et al., 2018) and mucilage from Arabidopsis seeds (Poulain et al., 2019). There is also an RG II region that is more complex but less frequent in general (Caffall & Mohnen, 2009), accounting for between 1% and 4% of the primary cell walls of dicots, nongrass monocots, and gymnosperms (O'Neill et al., 2004).

It is widely accepted that the technological functionality of pectin is primarily determined by the HG region (Rolin et al., 2010; Sousa et al., 2012). Critical parameters for HG functionality have been reported as the degree of methyl esterification and the distribution of charge within the HG region (Guillotin et al., 2005; Luzio, 2003; Willats et al., 2001). As indicated by Rolin et al. (2010) and Sousa et al. (2012), maximizing intrinsic viscosity ([*ƞ*]) also is an important consideration. Rolin et al. (2010) showed that using commercial pectin extraction methods on citrus fruit peel produced pectin with [*ƞ*] values ranging between 4 and 8 dl/g. Pectin functionality has a potential impact in many food, nonfood, and industrial/manufacturing applications related to rheology (Shah & Asadi, 2000), encapsulation (Gupta et al., 2012; Sun et al., 2019), texture modification (Schalow et al., 2018), charge capture (Dymińska et al., 2013; Gong et al., 2012), or water retention (Kieserling et al., 2019; Sharma et al., 2017). However, nonfood and industrial/manufacturing uses for pectin remain largely undeveloped (Endress & Williams, 2011).

The most common method for extracting commercial grade pectin is via the use of a hot acid process (Rolin et al., 2002). More recently, numerous alternative technologies have been proposed for pectin extraction, but none have been commercialized (Adetunji et al., 2017; Fishman et al., 2006; Kaya et al., 2014). We have developed a continuous process to release pectic hydrocolloids from their intracellular entrapment using steam explosion (Cameron et al., 2016, 2017a, 2017b; Dorado et al., 2017), which has the benefit of enabling pectin extraction from steam‐exploded citrus fruit peel using a simple water wash. Data from these previous studies suggest that the observed low *M*_w_ and [*ƞ*] may have resulted from the processing variables of temperature and time‐at‐temperature. To test this hypothesis, we utilized a static, batch steam explosion system (Grohmann et al., 2013) to process a stabilized lime pectin peel at temperatures ranging from 120 to 150°C and for hold times of 1, 2, and 3 min. Data on the recovery of the major pectic sugars, macromolecular properties, and functionality of the recovered pectic hydrocolloids were obtained.

## 2 MATERIALS AND METHODS

### 2.1 Steam explosion

Stabilized lime pectin peel (milled, washed, pressed, and dried peel from juice‐extracted fruit) was provided by CP Kelco. The stabilized pectin peel was rehydrated overnight with deionized water at 4°C using six weight equivalents of the peel mass. Steam explosion on 600 g of rehydrated pectin peel was performed with a static bench‐scale system (Dorado et al., 2019; Grohmann et al., 2013; Widmer et al., 2010). Steam was introduced into the reaction vessel, and the pressure was maintained to produce temperatures of 120, 130, 140, and 150°C by a thermocouple. The temperature is monitored using a McMaster‐Carr type thermocouple attached to a stainless steel cap. The thermocouple is inserted into the vessel at the top of the steam gun. Once the thermocouple is fully inserted, the cap and a silicone gasket at the top of the thermocouple are secured with a clamp to the steam gun. The thermocouple is attached to a custom‐built temperature monitoring box with a digital display. After obtaining the desired temperature and pressure, which took approximately 45 s (Grohmann et al., 2013), the pressure and temperature were maintained for 1, 2, or 3 min. Three replicates were performed for each treatment, and the three replicates were then pooled. The pooled samples were transferred to plastic bags, sealed, and stored at −20°C until analyzed.

### 2.2 Extraction of pectic hydrocolloids from steam‐exploded pectin peel

Pectic hydrocolloids were extracted from frozen, steam‐exploded pectin peel as previously described (Cameron et al., 2016). Briefly, equal masses of water and steam‐exploded tissue (100 g each) were mixed together and placed on a wrist shaker for 30 min. Two replicates were prepared for each treatment. A total of three washes were performed on each replicate. After each wash, the mixture was centrifuged at a relative centrifugal force (RCF) of 15,000 *g* for 20 min at 4°C. Following centrifugation, the wash liquids for each replicate were pooled and residual insoluble solids were removed from the supernatants by filtration through 1.2‐μm glass filter fiber (GF/C, Whatman/GE Healthcare Life Sciences Ltd.). An aliquot of the pooled supernatant was enzyme‐digested, and the pectic sugars present were quantified by HPAEC‐pulsed amperometric detection as previously described (Cameron et al., 2016). The percent recovery was calculated by dividing the amount of the sugar in the steam‐exploded biomass by the amount of the sugar in the pooled water extract. All estimates were made on a dry weight basis. Pectic hydrocolloids were recovered by precipitation with acidified ethanol at 4°C overnight (Cameron et al., 2016; Kertez, 1951). Following centrifugation, as described above, the pellets were frozen in liquid nitrogen and lyophilized as previously described (Cameron et al., 2016). Lyophilized pectic hydrocolloids were made into 2% (w/v) solutions in deionized water and then extensively dialyzed against multiple changes in deionized water using 6,000–8,000 Da MWCO dialysis tubing (Spectra/Por) overnight. After dialysis, the retentate was precipitated and lyophilized as previously described (Cameron et al., 2016). Dialyzed and lyophilized pectic hydrocolloids were stored at −80°C.

### 2.3 Recovery of pectic hydrocolloids

Percent recovery of the major citrus pectic hydrocolloid sugars (GalA, rhamnose, galactose, and arabinose) was estimated by the concentration present in the steam‐exploded peel and the recovered pectic hydrocolloids. The concentrations of these pectic sugars were estimated as previously described following enzymatic hydrolysis and high‐performance anion‐exchange chromatography (Cameron et al., 2016).

The DM of the pectic hydrocolloids was determined by titration according to a method modified from that found in the United States Pharmacopeia (Pharmacopeia US, 1995). The prepared solution of pectic hydrocolloids was titrated against sodium hydroxide of known molarity using bromothymol blue as an indicator and saponified for 15 min at room temperature with an excess of base. After the excess base was neutralized, the solution was titrated a second time, and the DM was calculated as in Equation (1).

(1) $$\text{DM}\;=\;\left(\frac{\text{moles}\;\text{methyl}‐\text{esterified}\;\text{GalA}}{\text{moles}\;\text{total}\;\text{GalA}}\right)\;\times \;100$$

### 2.4 High‐performance size‐exclusion chromatography of pectic hydrocolloids

Dialyzed, lyophilized pectic hydrocolloids were chromatographed as previously described (Dorado et al., 2019). A dn/dc value of 0.132 was used (Fishman et al., 2003). Electronic outputs from all the scattering angles measured by the multiangle light scattering detector (MALLS), differential pressure detector (DP), and differential refractive index detector (dRI) were processed with ASTRA software (Ver. 6.1.1.17; Wyatt Technology). Each sample was replicated a minimum of three times. The Astra software enables the estimation of several macromolecular parameters, including *M*_w_, *M*_n,_ and [*η*].

### 2.5 Functionality of recovered pectic hydrocolloids

#### 2.5.1 Standard acid in glass

USA‐SAG (standard acid in glass) values were determined using a Ridgelimiter according to methods detailed by the International Pectin Producers Association (Anonymous, 2017; Cox & Higby, 1944; Joseph & Baier, 1949). Gels were formulated assuming 150 °SAG. Briefly, gels are prepared to contain 650 g of total soluble solids (sucrose plus pectin). Assuming a 150 °SAG, the amount of pectin added would equal 650/150 = 4.33 g pectin, which is rounded down to 4 g, plus 646 g sucrose. The pectin is mixed with 20–30 g of the sugar and solubilized in 410 ml deionized water. After solubilization of the sugar plus pectin, the solution is heated to boiling, and the remaining sugar is added in two portions. Then, the solution is heated until a weight of 1,015 g is reached. The heated solution is allowed to rest for 1 min and then poured into prepared glass cups of standardized size and shape, which contain 2 ml of a 48.8% (w/v) tartaric acid solution. The jellies are then stored for 20–24 hr at constant temperature (25°C ± 3°C) before being removed from the glass. The amount of sag is measured after 2 min using a Ridgelimiter.

#### 2.5.2 Rheology

Sugar–acid gels made from recovered pectic hydrocolloids were prepared by the method of Yoo et al. (2003) with only slight modifications. Pectic hydrocolloids (0.2 g) were solubilized in 7.3 g of 0.1 M citrate buffer (pH 3.0) by stirring overnight. Subsequently, they were centrifuged for 30 min at an RCF of 12,100 *g*. The supernatant was brought to 60% sugar, which was dissolved thoroughly in a 98°C water bath for 30 min. The sugar gel was placed on the Peltier of the rheometer (AR1000; TA Instruments), and the geometry (parallel plate, 500 µm gap) was lowered into place. The excess gel which extruded from under the geometry was removed. Gel conditioning was done at 20°C for 2 min prior to measuring the rheological properties of the pectin–sugar mixtures in dynamic shear. Dynamic shear data were obtained from frequency sweeps over the range of 0.08–628 rad/s at a 2% strain, which was in the linear viscoelastic region. Storage modulus (G′) and loss modulus (G″) were obtained, and tan δ, which indicates more gel‐like than liquid‐like properties, was calculated dividing G″ by G′.

### 2.6 Statistical analysis of data

An optimization analysis was performed using Design‐Expert (version 11.0.5.0). The adequacy of the model was determined by evaluating the lack of fit, coefficient of regression (*R* ^2^), and the *F*‐value obtained from the analysis of variance (ANOVA). Statistical significance of the model and model variables was determined at the 95% confidence level (*p* < .05). Other statistical analyses were performed using either Excel (Microsoft Office 2016), GraphPad Prism (version 4.3), or Design‐Expert (version 11.0.5.0).

## 3 RESULTS AND DISCUSSION

### 3.1 Recovery of pectic hydrocolloids from steam‐exploded pectin peel

**FIGURE 1:**
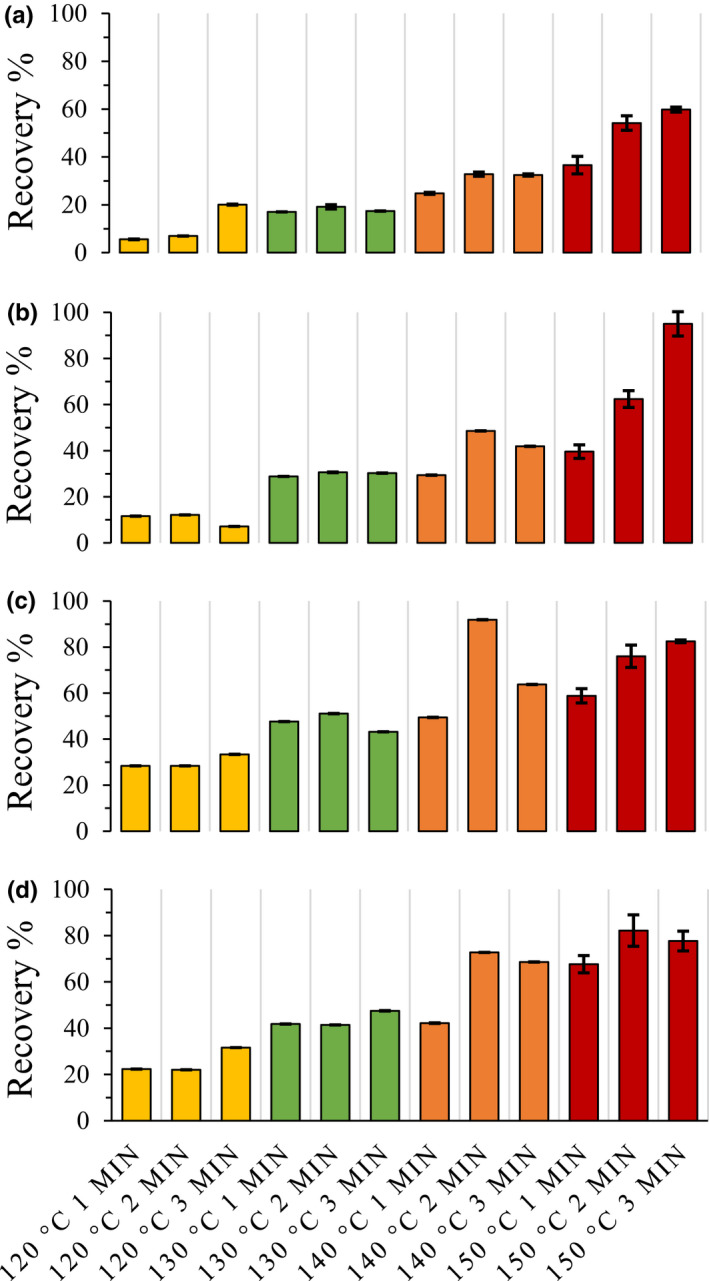
The recovery percentage of major pectic sugars from steam‐exploded citrus peel. (a) Rhamnose; (b) galactose; (c) arabinose; (d) galacturonic acid

The measured recovery percentage of the major pectic sugars generally increased with increasing temperature and time‐at‐temperature (Figure 1). GalA recovery, the dominate sugar in citrus pectin, ranged from 22% to 82% (Figure 1d). The response surface and contour plot illustrating the predicted relationship between time and time‐at‐temperature for GalA recovery percent from steam explosion, indicates that time, time‐at‐temperature, or both can be manipulated to obtain desired levels for GalA recovery (Figure S1A). For percent recovery of GalA, ANOVA indicated that the quadratic model was significant at *p* = .01 level. Only temperature was a significant factor at the *p* = .001 level (Table 1).

**TABLE 1:** ANOVA statistics for results that demonstrated a significant (*p* > .05) statistical model

	% Recovery (GalA)			[*η*] dl/g			G′		
	*F*	*p*	*R* ^2^ (%)	*F*	*p*	*R* ^2^ (%)	*F*	*p*	*R* ^2^ (%)
Model	42.54	***	90.43	42.38	***	94.08	18.81	**	65.29
X_1_	81.01	***		104.87	***		18.81	**	
X_2_	4.07			8.22	*				
$X_{1}^{2}$				14.05	**				

Note

The models were reduced by removing terms with *p* values > .1. [*η*] = intrinsic viscosity (dl/g); G′ = storage modulus; *p* = probability value; *R* ^2^ = regression coefficients. *, **, and *** represent significance at *p* < .05, *p* < .01, and *p* < .001, respectively; X_1_ and X_2_ represent temperature (°C) and time‐at‐temperature (min), respectively.

Previous results from either continuous or single‐batch steam explosion of citrus peel produced recovery percentages (based on GalA) ranging between 58% and 78% for raw, unwashed orange fruit peel (Cameron et al., 2016; Grohmann et al., 2013). At a treatment temperature of 160°C, Grohmann et al. (2013) observed a decrease, from 89% to 59% in GalA recovery percent with increased time‐at‐temperature up to 4 min. A reduction in recovery of GalA was also observed in raw Valencia peel with increasing time‐at‐temperature at 170°C (Dorado et al., 2019). Dorado et al. (2019) also reported an increase in GalA recovery for raw Hamlin fruit peel at 130°C as time‐at‐temperature increased and that recovery decreased with time‐at‐temperature at 150 and 170°C. Here, we saw a general increase in recovery percent with increased time‐at‐temperature up to 150°C (Figure 1d). The continued increase in GalA recoveries at elevated temperatures observed here may be a result of the peel having been washed, to remove soluble sugars, prior to drying. The abovementioned previous studies all used unwashed fruit tissue which contained significant amounts of soluble sugars.

### 3.2 Macromolecular characterization of recovered pectic hydrocolloids

#### 3.2.1 Weight average molecular weight

**FIGURE 2:**
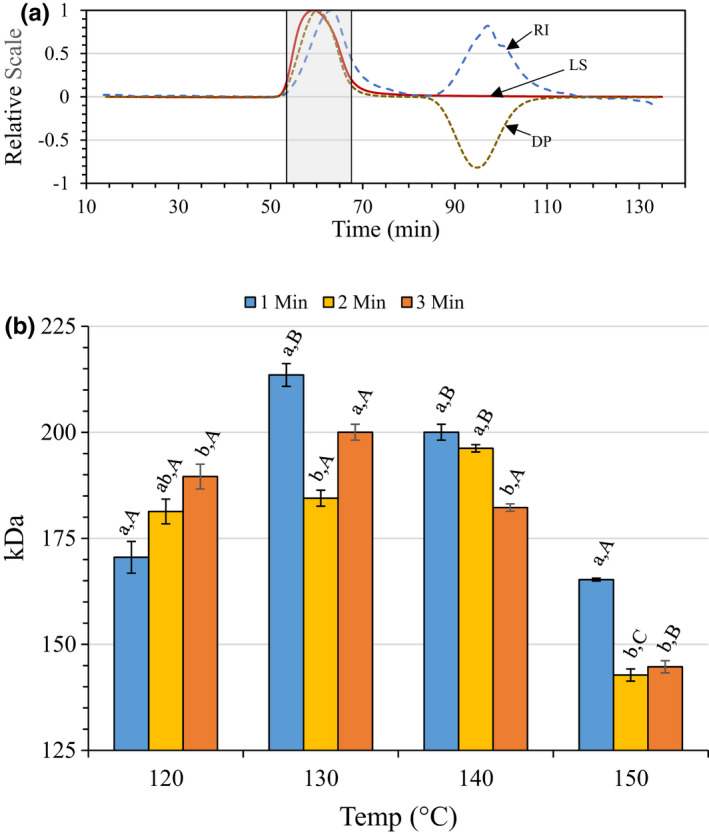
(a) Representative SEC‐MALLS‐RI‐DP chromatogram. The shaded box represents the area of the chromatogram designated as the pectic hydrocolloid peak. LS = laser, RI = refractive index, DP = differential pressure. (b) Weight average molecular weight of recovered pectic hydrocolloids. Error bars represent the standard error of the mean for of at least three replicates. Bars with different lower case letters indicate a statistically significant difference (*p* > .05) within a temperature group, and bars with different upper case letters indicate a statistically significant difference (*p* > .05) for different temperatures within a treatment time

Figure 2a illustrates a representative chromatogram from SEC‐MALLS chromatography, indicating the area designated as the pectic hydrocolloid peak. Figure 2b shows that *M*_w_ increased with increasing temperature or time‐at‐temperature up to 130°C‐1 min and then decreased until reaching a low point at 150°C‐2 min. The range in *M*_w_ was 142–214 kDa.

In general, *M*_w_ is thought to be an important element in commercial pectin functionality, but it may vary widely between 100 and 400 kDa, and greater, depending on the type of citrus being used (Fishman et al., 2003; Kaya et al., 2014; Rolin et al., 2002, 2010; Sayah et al., 2016). Grohmann et al. (2013) also saw a decrease in *M*_w_ at 160°C with increased time‐at‐temperature. In contrast, Dorado et al. (2019) observed a gradual increase in *M*_w_ with increasing temperature and time‐at‐temperature for Valencia fruit peel and a more dramatic increase with Hamlin fruit peel. It must be noted, however, that the Grohmann et al. (2013) and Dorado et al. (2019) (McNeil et al., 1984) used raw, unwashed peel, while in this study a commercial washed and dried pectin peel was utilized.

#### 3.2.2 Degree of methylesterification

**FIGURE 3:**
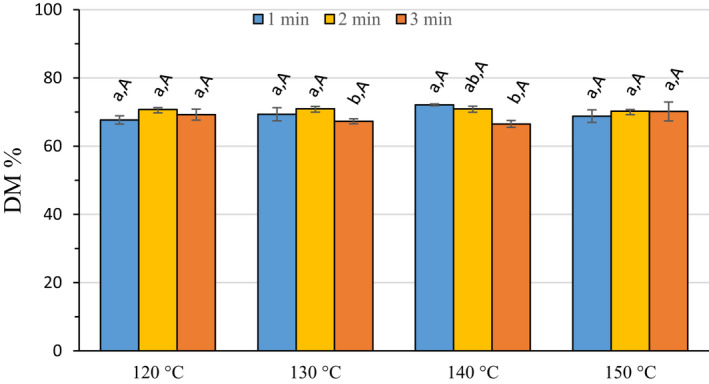
Degree of methylesterification of recovered pectic hydrocolloids. Error bars represent the standard error of the mean of at least three replicates. Bars with different lower case letters indicate a statistically significant difference (*p* > .05) within a temperature group, and bars with different upper case letters indicate a statistically significant difference (*p* > .05) for different temperatures within a treatment time

There were significant differences among DM values with the various treatments, for both time‐at‐temperature within a temperature group and for identical times between temperature groups (Figure 3). Despite these statistically significant differences in DM, it is unlikely that a functional difference due to DM would be observed in any of these pectins, since the DMs fell within a limited range (64.39–76.81) with a grand mean of 69.53 and a standard deviation of 2.76. All of these pectic hydrocolloids would be classified as a high DM pectin since the DM was >50%. High DM pectins are used for applications where low pH and added sugar facilitate gelation (May, 1990).

### 3.3 Functionality of recovered pectic hydrocolloids

#### 3.3.1 Intrinsic viscosity

**FIGURE 4:**
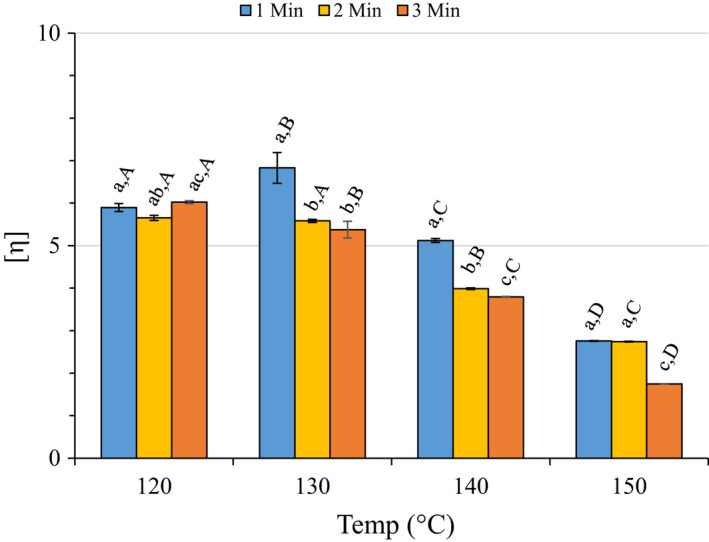
Intrinsic viscosity ([*η*]; dl/g) of recovered pectic hydrocolloids. Error bars represent the standard error of the mean of at least three replicates. Bars with different lower case letters indicate a statistically significant difference (*p* > .05) within a temperature group, bars with different upper case letters indicate a statistically significant difference (*p* > .05) for different temperatures within a treatment time

Functionality, as measured by intrinsic viscosity [*η*], was significantly affected by the experimental parameters (Figure 4, Table 1). The model was significant at *p* = .001, temperature was significant at *p* = .001, time‐at‐temperature was significant at *p* = .05, and temperature^2^ was significant at *p* = .01. Two‐way ANOVA revealed numerous significant differences within a temperature group for the various times and between temperature groups for time‐at‐temperature (Figure 4).

Figure S1B is the response surface and contour plot illustrating the predicted relationship between time and time‐at‐temperature for [*ƞ*]. [*ƞ*] held reasonably constant throughout the 120 and 130°C treatments, although [*ƞ*] for the 130°C‐1 min treatment was significantly greater than the other 120 or 130°C treatments (Figure 4). [*ƞ*] began to decline after the 140°C‐1 min treatment and reached its lowest value at 150°C‐3 min. Rolin et al. (2010) indicated that pectin extracted from citrus peel under commercial extraction methods had [*ƞ*] values between 4 and 8 dl/g.

#### 3.3.2 Rheology

**FIGURE 5:**
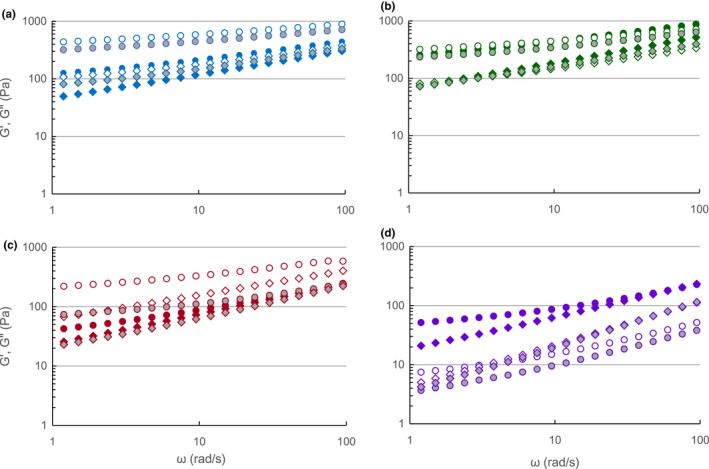
Dynamic mechanical storage modulus (G′ in Pa) and loss modulus (G″ in Pa) for sugar–acid gels. (a) 120°C, (b) 130°C, (c) 140°C, (d) 150°C. G′ = circle, G″ = diamond, tan δ = square. Closed = 1 min, open = 2 min, shaded = 3 min

The functionality of the recovered pectic hydrocolloids also was explored by examining the rheology of sugar–acid gels. Figure 5 shows changes in storage modulus (G′; a measure of the energy stored in the material or recoverable per cycle of deformation) and loss modulus (G″; a measure of the energy lost as viscous dissipation per cycle of deformation) as a function of the frequency (ω) for pectin–sugar mixture samples at 20°C. From 120 to 140°C, all pectin–sugar gels exhibited G′ higher than G″, which represented more viscoelastic, gel‐like properties than liquid‐like properties. However, the 140°C‐3 min gel displayed a tan (δ) of 0.98 (Figure S2). Values greater than 1.0 represent a more viscous property than viscoelastic property, within the range of shear thinning.

Tan (δ) quantifies the balance between energy loss and storage. A value for tan (δ) greater than unity indicates more liquid properties, whereas one lower than unity means more solid properties, regardless of the viscosity. Samples prepared at 150°C for 2 and 3 min manifested more viscous properties than viscoelastic, gel‐like properties which were indicated by tan (δ) values higher than 1.0 (Figure S2). The loss of gelling properties in these samples could be derived from their lower *M*_w_ compared with samples from other treatments. For G′, the ANOVA model was significant at *p* = .05, and temperature was a significant factor at *p* = .01 level (Figure S1C and Table 1), manifesting higher G′ at lower temperatures.

The magnitudes of G′ and G″ of pectin–sugar mixtures increased as ω increased, showing that G′ was much higher than G″ at all values of ω with high frequency dependency. A similar trend has been reported with other high DM pectin containing sugar gels (Evageliou et al., 2000; Silva et al., 1995). Plots of ln G′ and G″ versus ln ω of true gels typically displays a slope of zero, and G′ is higher in magnitude than G″ over broad ranges of ω with moduli almost parallel to each other (Ross‐Murphy & Chan, 1984) while weak gels have positive slopes. The strong dependence of G′ and G″ on ω with positive slopes clearly showed that all pectin–sugar mixture samples exhibited behavior typical of weak gels. Such behavior of mixtures can be related to the role of sugars in the formation of the elastic gel network of pectin by associating with pectin molecules via hydrogen bonding to form secondary links that reinforce the molecular network as indicated by Nussinovitch (1997).

#### 3.3.3 Standard acid in glass (SAG)

**FIGURE 6:**
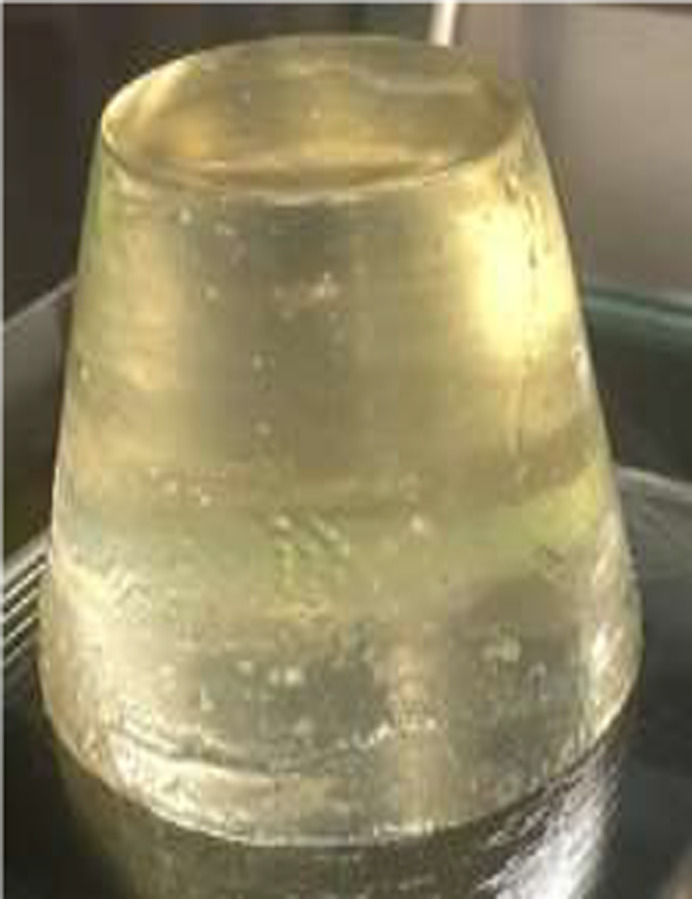
Gel produced from pectic hydrocolloids recovered from the 120°C‐2 min treatment used for °SAG determination

Functionality, as based on USA‐SAG testing, was limited as pectic hydrocolloids from the 120°C‐3 min, and all of the 130°C treatments did not produce a gel that could be measured using the Ridgelimiter. The °SAG could only be measured on the 120°C‐2 min gel (Figure 6) as there was insufficient material to test the 120°C‐1 min pectic hydrocolloids. The 120°C‐2 min gel had a °SAG value of 180. Since the 130°C samples did not form sufficiently strong gels, the 140 and 150°C samples were not tested.

### 3.4 Optimization analysis for intrinsic viscosity and percent recovery

Using the historical data capabilities of the Design‐Expert software, we performed an optimization analysis to estimate the optimal temperature and time‐at‐temperature to maximize both [*ƞ*] and percent recovery. The goal for temperature was set within the range, and time, [*ƞ*], and % recovery were set to maximize their value. Time was maximized because longer time‐at‐temperature values are more realistically feasible than very short ones. The models were reduced by removing terms with *p* values greater than *p* > .1 as recommended by Design‐Expert. The Desirability function using these parameters was 0.708. Using coded values (−1, 0, +1, etc.) for time and time‐at‐temperature, the equation for predicting [*ƞ*] was:

(2) $$\left[\eta \right]\;=\;5.24\;‐\;1.79\;\times \;\text{temperature}\;‐\;0.46\;\times \;\text{time}\;‐\;1.10\;\times \;{\text{temperature}}^{2}$$

and for predicting percent recovery, it was:

(3) PercentRecovery=47.91+23.27×temperature+4.76×time

**FIGURE 7:**
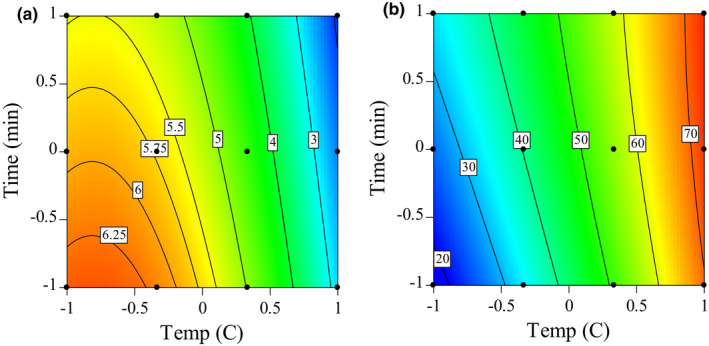
Predicted intrinsic viscosity (a) and % recovery (b) using optimization analysis while maximizing both pectic hydrocolloid recovery percent and intrinsic viscosity. Axis values are coded values where −1 = 120°C, 0 = 135°C, and 1 = 150°C for temperature and −1 = 1 min, 0 = 2 min, and 1 = 3 min for Time. Contour lines represent intrinsic viscosity (a) or % recovery (b)

Contour plots for the optimization analysis (Figure 7) show the surfaces resulting from these equations. Figure 7a shows the effect of time and time‐at‐temperature on [*ƞ*], and Figure 7b illustrates the effects on percent recovery. These results indicate that depending on the functional properties desired, the percent recovery can either be maximized up to about 75% with an [*ƞ*] of under 3.0 or [*ƞ*] can be maximized with a reduced recovery. However, there are numerous solutions where an [*ƞ*] between 4.0 and 6.0, and with recoveries approximating 50%, can be obtained with a simple water wash.

## 4 CONCLUSIONS

The results presented here and by Dorado et al. (2019) (Widmer et al., 2010) support our hypothesis that temperature and time‐at‐temperature used in our previous studies (Cameron et al., 2016, 2017b; Grohmann et al., 2013) likely were responsible for the low *M*_w_ and [*η*] of the recovered pectic hydrocolloids. In a study on the hydrothermal production of uronic acids using pectin as a model substrate, Pińkowska et al. (2019) saw a decrease in uronic acid yield with increasing temperature, and their transformation into furfurals. Although hydrothermal treatment does not have the decompression impact that steam explosion has, Muzamal et al. (2015) has shown that the explosion step alone is not necessary to disintegrate the biomass studied. Brownell et al. (1986) also showed that pressure drop itself was not necessary for the release of glucose. These results also demonstrate that manipulating either temperature or time‐at‐temperature would enable the production of structurally varied populations of pectic hydrocolloids. Using steam explosion coupled to simple water extraction to obtain pectic hydrocolloids from juice‐extracted or culled citrus fruit could open new applications for inexpensive, environmentally friendly pectic hydrocolloids where rheology modification, ion‐capture, or hydration control functionalities are needed.

## Supporting information

Figure S1

Figure S2

## Data Availability

Data available on request from the authors. The data that support the findings of this study are available from the corresponding author upon reasonable request.
