# Supplementary material for: Pectic hydrocolloids from steam‐exploded lime pectin peel: Effect of temperature and time on macromolecular and functional properties
Source: Food Sci Nutr. 2021 Feb 12;9(4):1939–48. doi: 10.1002/fsn3.2158 (PMC8020944; doi:10.1002/fsn3.2158)
Supplement: Supplementary file 1 — Figure S1 [file FSN3-9-1939-s001.docx]

Figure S1. Response surfaces and contour plots for: (A) percent recovery of Galacturonic Acid; (B) [ƞ] dL∙ g^-1^; (C) G′ of pectic hydrocolloids. Dark and light dots are the experimental values. Axis values were coded for Temperature -1 = 120 °C, 0 = 135 °C, 1 = 150 °C; for Time -1 = 1 min, 0 = 2 min, 1 = 3 min.

G'

[η]

% Recovery

**B**.

**C**.

**A**.

Time (min)

Time (min)

Temperature (ͦ C)

Temperature (ͦ C)

Time (min)

Temperature (ͦ C)
