# Supplementary material for: Pectic hydrocolloids from steam‐exploded lime pectin peel: Effect of temperature and time on macromolecular and functional properties
Source: Food Sci Nutr. 2021 Feb 12;9(4):1939–48. doi: 10.1002/fsn3.2158 (PMC8020944; doi:10.1002/fsn3.2158)
Supplement: Supplementary file 2 — Figure S2 [file FSN3-9-1939-s002.docx]

Figure S2. Tan δ for sugar acid gels. (A) 120 °C, (B) 130 °C, (C) 140 °C, (D) 150 °C. Closed = 1 min, open = 2 min, shaded = 3 min.

B

A

D

C
